# The effect of new LED lighting systems on the colour of modern paints

**DOI:** 10.1038/s41598-021-01836-9

**Published:** 2021-11-17

**Authors:** Valentina Pintus, Ferenc Szabó, Renáta Gazdag-Kéri, Dávid Noel Tóth, Róbert Nagy, Péter Csuti, Katja Sterflinger, Manfred Schreiner

**Affiliations:** 1 Institute of Science and Technology in Art, Academy of Fine Arts, Schillerplatz 3, 1010 Vienna, Austria; 2 Institute for Conservation-Restoration, Modern-Contemporary Art, Academy of Fine Arts, Schillerplatz 3, 1010 Vienna, Austria; 3 Light and Colour Science Research Laboratory, Department of Electrical Engineering and Information Systems, Faculty of Information Technology, University of Pannonia, Egyetem Str., Veszprém, Hungary

**Keywords:** Chemistry, Materials science

## Abstract

This research focuses on the investigation of the effect of a new light emitting diode (LED)-lighting system which reproduces indoor museum conditions, on some self-made art paint colours (acrylic-, alkyd-, and linseed oil-based paints) often used in modern-contemporary art. A halogen lamp representing a traditional light source for museum lighting was also considered. Lighting-set-up and lighting optimization parameters were considered while Ultraviolet/Visible/Near Infrared (UV/Vis/NIR) spectrophotometry was used for investigating the colour change of the paint samples. Univariate analyses allowed determining the highest effect of the lighting systems on the linseed oil binder and ultramarine blue PB29 mixture upon ageing, according to the highest total colour change ΔE*_ab_. In a more specific and detailed way, variance analyses not only demonstrated the strong correlation between the type of binder and pigment used for the paint samples with the colour variation, but also showed that the short-wavelength blue LEDs influenced the change along the yellow–blue b* axis of the yellow and blue samples, whereas the halogen lamp mostly had an impact towards the red–green a* axis of mostly the green specimens.

## Introduction

Modern-contemporary art is an increasingly important part of museum collections around the world, and thus its preservation and conservation has become a fundamental task. Unfortunately, they have been observed to undergoing ageing and degradation processes in a relatively short time in comparison to traditional art materials. Because they are typically made with materials with a very complex chemical composition, and then kept in a wide variety of environmental conditions, these processes are not well understood. One of the main environmental parameters concern in indoor museum is the light or lighting system. Indoor museums exhibiting vast group of modern-contemporary art objects, especially paintings, need proper illumination systems to provide the best possible viewing conditions and a comfortable atmosphere for visitors, while avoiding the harmful effects of optical radiation on the artworks. Modern light sources such as light emitting diode (LED)-based ones, which belong to the group of solid-state lighting (SSL), can be found nowadays in an increasing number of applications in museums and several research institutions are developing guidelines regarding their selection^1,2^. Although the LEDs are being increasingly implemented in indoor museums more and more, the long-term effects of their emitted light output on artworks are not yet well known. Therefore, LED-based lighting systems and their effects on the stability of paint materials used in art still needs to be studied in order to determine if they can be potentially detrimental to the different types of components usually included in a paint system, for example by fast photo-oxidative deterioration. Additionally, although the number of LED ageing studies on paint materials are increased over the last few years^3–12^, a comprehensive research focused on the investigation of the chemical stability of the irradiated materials is still lacking.

### Comparison to incandescent light in indoor museums

In comparison to light sources traditionally found in museums such as halogen incandescent lamps, LEDs have a different illumination source, as well as working principles. For instance, while incandescent light sources produce light through the heating of a filament, LEDs emit light under the electroluminescence principle. This is based on the recombination of electrons with electron holes in a two-lead semiconductor source as a p–n (positive–negative) junction diode, when a suitable voltage is applied. Based on this working principle, LEDs need very little power for the same luminous flux output as a halogen incandescent lamp, making them far more efficient in energy consumption and output. Besides their differing functioning principles, the luminous efficacy of LEDs and incandescent lamps is very different. This is a measure of how efficient a light source produces visible light and is defined as the ratio of the luminous flux as the perceived power of light (lumens—lm) to power (Watt—W). Luminous efficacy of LEDs is considerably higher than traditional incandescent lamp; up to 150 lm/W for LEDs in contrast to a maximum of 26 lm/W for the incandescent lamps^1^. Moreover, the most important advantage of LEDs in comparison to traditional incandescent lights is that they emit no radiation in the damaging UV or infrared ranges. The UV range has already been demonstrated to be harmful for the stability of paint materials^13–17^. Thus, due to their high luminous efficacy, long lifetime and reliable operating characteristics LEDs are becoming more prominent in indoor museums and archives, while the incandescent lighting solution has been gradually pushed into the background. Especially, the manufacturing, importation, sale of incandescent lamps for general lighting has been banned by a phase out regulation emended by governments around the world, for favouring more energy-efficient lighting alternatives. For instance, European Union phased them from 2009 to 2012, while in the United States the state of California introduced legislation in 2007 to phase out the use of incandescent bulbs by 2018.

### The damaging effect of light on art materials in indoor museums

The field of art is characterized by a vast group of different objects, especially represented by paintings. The materials used for paintings can be organic or inorganic, and the type of pigment and binder contained in the colour can vary from natural organic or synthetic organic depending on the historical period in which it was employed. The ageing chemical behaviour of a paint material is related to its chemical composition and particularly to the environment in which it is kept. Indoor conditions such as museums, where an enormous number of paintings are usually displayed, may influence the speed of their degradation depending on some chosen parameters such as temperature, relative humidity as well as light exposure. It is already well known that with increasing photon energy (shorter wavelength of optical radiation—blue and UV range) the probability of photochemical reactions increases, which may contribute to colour shifts of paints. Therefore, the investigation of art materials when exposed to lighting system such as LEDs is fundamental for understanding their chemical stability and for developing suitable conservation strategies.

To avoid the harmful effect of light sources a limitation of illuminance (lux level in the SI system, 1 lx = 1 lm per square meter)—that correlates with the intensity of the visible light—is recommended in indoor museums, depending on the type of exposed materials. This parameter might variate from a minimum of 50 lx for highly sensitive items such as textiles to a maximum of 300 lx for less sensitive ones like stone, ceramic, glass and metals^18^. The Commission Internationale de l´Eclairage (CIE) published the CIE 1990^19^ and CIE 2004^20^ guidelines for the illumination of different artworks in which art conservation aspects are also discussed besides visual aspects. According to these guidelines, the colour changes of light sensitive materials depend on irradiation and spectral power distribution of the lighting, spectral responsivity of the given material, and duration of exposure. Particularly, the CIE 2004 covers both the heating effects and photochemical effects on the materials, and enumerates the relative damage potential of different light sources by giving a formula on the calculation of the damage potential. Nevertheless, these guidelines are far too old to consider LED lighting as an alternative for museum lighting, and are mainly applicable to traditional light sources. Additionally, the estimation of the harmful effects of a light source on art materials should also consider the intrinsic chemical properties of the irradiated material, which can be studied through the use of meaningful scientific methods.

### Aim of the research

This research presents important information obtained through a series of experiments for the investigation of the damaging effects of new developed LED lighting systems on modern paint materials. This work aims to report the fundamental arrangement of the new developed tuneable LED lighting systems and halogen lamp for indoor museums into suitable chambers as well as the optimization of the lights parameters. Furthermore, in order to determine any changes caused by exposure to the different illumination systems, both to the optical appearance of the paint material (e.g., colour changes)—as well as in the paint structure (e.g., chemical changes), a multi-analytical approach was developed. More precisely, the investigations of colour change, the first recognizable indications of the harmful impact of the lighting system on paint surface, were performed by Ultraviolet/Visible/Near Infrared (UV/Vis/NIR) spectrophotometry and reported in this work. The chemical stability of paint materials, which was investigated by Pyrolysis–Gas Chromatography/Mass Spectrometry (Py–GC/MS), Thermally assisted Hydrolysis and Methylation of GC/MS (THM-GC/MS), micro-Attenuated Total Reflection of Fourier Transform Infrared (µ-ATR-FTIR), and µ-Raman spectroscopies, will be the subject of a near future publication.

## Results and discussion

### Lighting exposure conditions and set-up

The operating conditions of the three used lighting systems, the LEDs with the two different blue wavelengths peaks (420 nm—named as LED A, and 460 nm—named as LED B) and the incandescent halogen lamp—are hereby described and additionally summarized in Table 1. Within this context two main different aspects of the light sources are considered are discussed in the “Methods” section: (1) the apparent colour of the lighting—described by fundamental colour parameters—and (2) the ability of the light source to reveal the true colours of an object—defined by the colour rendition or colour fidelity metrics. On the other hand, the damage index (DI), critical duration of exposure (*t*_*s*_), and illuminance level (*E*_v_ [lx]) are here reported and were optimized for performing the accelerated ageing, thus simulating indoor museum light conditions over a long period of time. The building and set-up of three different lighting chambers necessary for accommodating and exposing the paint samples are highlighted in Supplementary Information. Additionally, the temperature and relative humidity conditions during the ageing are also considered.

**Table 1:** Parameters of the lighting systems used.

Lighting	*E*_v_ (lx)	*DI*	*t*_s_ (h)	CCT (K)	*d*_uv_	CRI (*R*_a_)	IES (*R*_f_)	IES (*R*_g_)
LED A (420 nm)	2797	0.181	1146	3677	− 0.0002	92	79.9	106.4
LED B (460 nm)	2788	0.138	1520	3655	0.0008	88	84.5	97.9
Halogen	2923	0.174	3532	3743	− 0.0054	93	96.2	102.8

*E*_v_ [lx]/damage index: *DI*/and critical duration of exposure in hours: *t*_s_. Discussed in methods section: CCT: correlated colour temperature in Kelvin (K)/CRI: colour rendering index in *R*_a_/Illuminating Engineering Society (IES) colour fidelity in *R*_f_/Illuminating Engineering Society (IES) gamut area index in R illuminance level in lux: lx.

#### Damage index (DI), critical duration of exposure (***t***_s_), and illuminance level (***E***_v_ [lx])

Table 1 lists additional parameters of the three lighting systems used for this work such as damage index (DI), critical duration of exposure in hours (*t*_s_), and illumination level (*E*_v_ [lx]), calculated according to CIE 2004 technical report^20^. These factors are very important for assessing the potential risk of optical radiation, which depends not only on the lighting system but also on the material of the illuminated object. The CIE 2004 enumerates the relative damage potential of different light sources except LEDs and discusses a procedure of the calculation of the damage potential. According to this, the Damage function *D*(*λ*) defines the relative spectral responsivity of a material and it is used to determine a Damage Index (*DI*) for incident radiation. Based on the calculation process, the relative (rel) damage flux (F_dm_) is given by Eq. (1):

1 $$F_{{{\text{dm}},{\text{rel}}}} = \mathop \smallint \limits_{{\left( {\uplambda } \right)}}^{{}} \Phi \left( \lambda \right) T\left( \lambda \right)D\left( \lambda \right) d\lambda$$

where *Φ*(*λ*) is spectral radiant power (W/nm), *T*(*λ*) is spectral transmittance of filter, *D*(*λ*) is damage function, *λ* is wavelength (nm).

And the relative luminous flux is obtained by the following formula as Eq. (2):

2 $$F_{{{\text{v}},{\text{rel}}}} = \mathop \smallint \limits_{{\left( {\uplambda } \right)}}^{{}} \Phi \left( \lambda \right)T\left( \lambda \right)V\left( \lambda \right)d\lambda$$

where V(*λ*) is the spectral luminous efficiency for photopic vision.

Then the Damage Index (*DI*) for the incident radiation can be thus calculated as Eq. (3):

3 $$DI = F_{{{\text{dm}},{\text{rel}}}} /F_{{{\text{v}},{\text{rel}}}}$$

Based on this damage calculation process, the determination of the *DI* is possible prior to the actual design of the museum lighting. Shortcomings of the presented CIE Publications are that these have not considered solid state lighting as museum illumination. On the other hand, within this research the calculated *DI* for the three used lighting systems was 0.181 for the LED A, 0.138 for LED B, and 0.174 for halogen lamp.

Another important factor is the *Threshold Effective Radiant Exposure H*_s,dm_, which is the amount of radiation energy that causes a visible change in the object´s colour (one unit of ΔE*_ab_ colour change), and this increases as damage progresses [Eq. (4)].

4 $$H_{{{\text{s}},{\text{dm}}}} = E_{{{\text{dm}}}} {*}t_{{\text{s}}} \;\;{\text{Wh/m}}^{{2}}$$

where *E*_dm_is the effective irradiance (W/m^2^), *t*_*s*_ is the critical duration of exposure hour.

**Figure 1:**
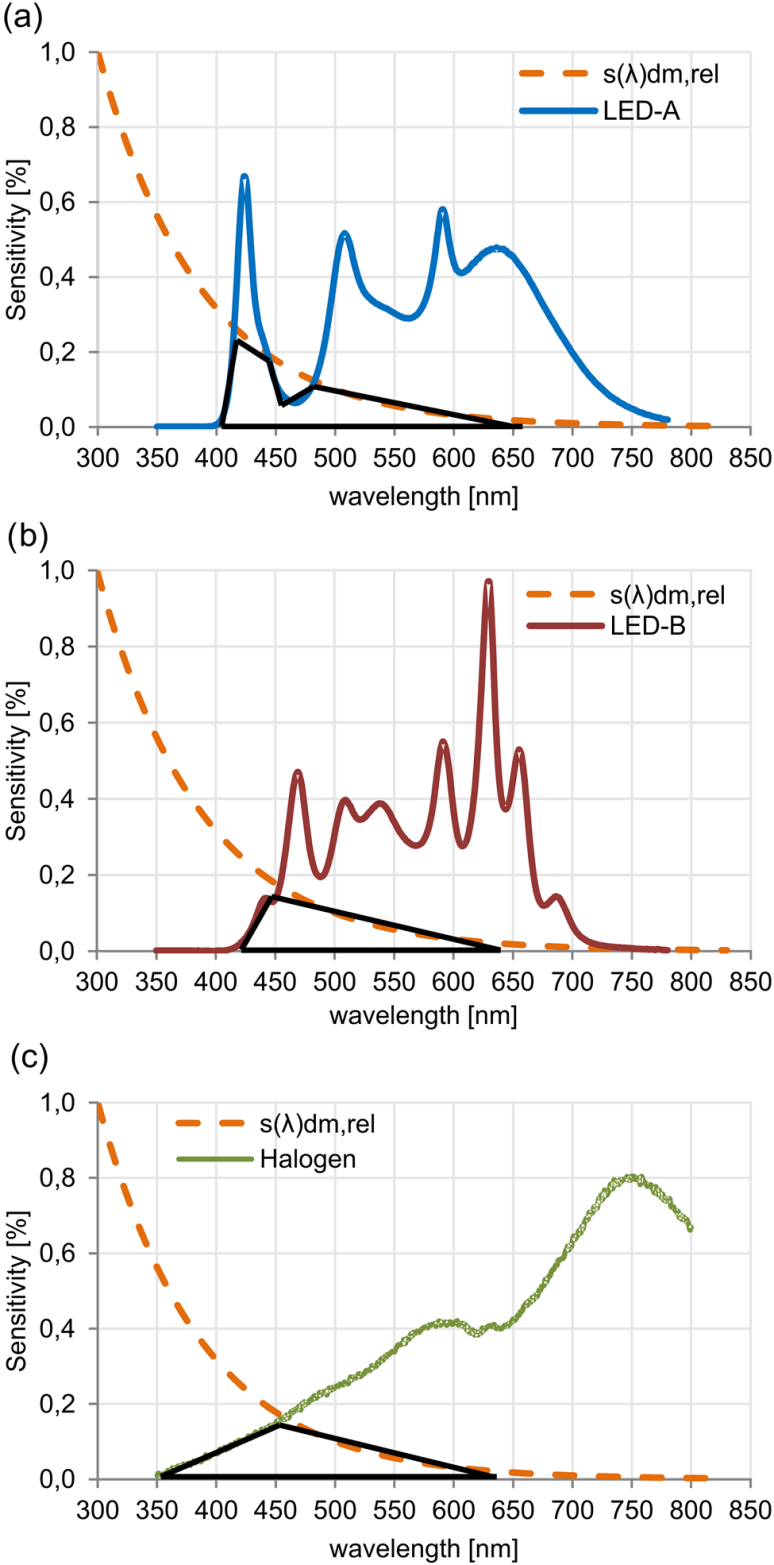
Harmful radiation content of the lightings depicted by the black triangle: (**a**) LED A (420 nm), (**b**) LED B (460 nm), and (**c**) halogen lamp.

In case of the three test lights *H*_s,dm_ was reached among 1250–5000 h irradiance (this means the critical duration of exposure—t_s_). Table 1 shows the calculated *DI* and *t*_s_ of the used lighting systems. Based on the calculation LED B spectrum (with maximum blue peak at longer wavelength blue—460 nm) means 20% lower damage load compared to the halogen lamp, while LED A (with maximum blue peak at short wavelength blue—420 nm) leads to an increase of only 4% higher damage above that of the incandescent halogen lighting. Figure 1 shows the harmful radiation range of the ageing spectra (highlighted with black), where *S*(*λ*)_dm,rel_ means the relative spectral responsivity of the object represented by the general simplified following form as Eq. (5):

5 $$S\left( \lambda \right)_{{\left( {{\text{dm}},{\text{ rel}}} \right)}} = \alpha \left( \lambda \right){*}f^{\prime}\left( \alpha \right)$$

Since α(λ) is nearly constant for many non-pigmented materials, it may be assumed that *s*(*λ*)_dm,rel_ = *f*′(*λ*). Data from periodic colorimetric measurements have indicated that *s*(*λ*)_dm,rel_ may be represented by an exponential function of the form^20^ as (6):

6 $$S\left( \lambda \right)_{{\left( {{\text{dm}},{\text{ rel}}} \right)}} = e^{{ - b\left( {{\uplambda } - 300} \right)}}$$

The *s*(*λ*)_dm,rel_ function defines the action spectrum for each category of materials with *material constant b.* 1 is its normalised value at a wavelength of 300 nm, since lights with radiation below this values rarely occur in indoor museums. In Fig. 1 it can be seen that the harmful radiation content is approximately equal in case of the LED A and the halogen incandescent lamp.

The uniformity of illumination, which was measured on several different points occupied by the samples prior the lighting exposure, was at least 0.98 in each ageing chamber, which is close to the optimal value of 1. Regarding the illumination level (*E*_v_ [lx]), museums’ curators prefer moderate values (50–300 lx) for art preservation reasons. For performing accelerated ageing an illuminance level of 3000 lx was calculated (Table 1) as the maximum level possible without exposing the samples to excessive heat. The samples were exposed for a total amount of 5000 h of irradiance period as the maximum threshold exposure or maximum critical radiation time *t*_s_. According to these lighting conditions, the exposure thus reproduced around 20.000—50.000 h of indoor museum lighting, depending on the spectral composition of the light sources.

### Colour investigation

The UV/Vis/NIR results reported here include the shift of colour coordinates and total colour change ΔE*_ab_ obtained by comparing the unaged and 5000 h aged samples (representative of noticeable colour changes) and by considering the wavelength range of 380–730 nm. For the obtained reflectance spectra in the UV and NIR range of the unaged and aged samples no significant variation could be observed.

#### Univariate analyses

**Figure 2:**
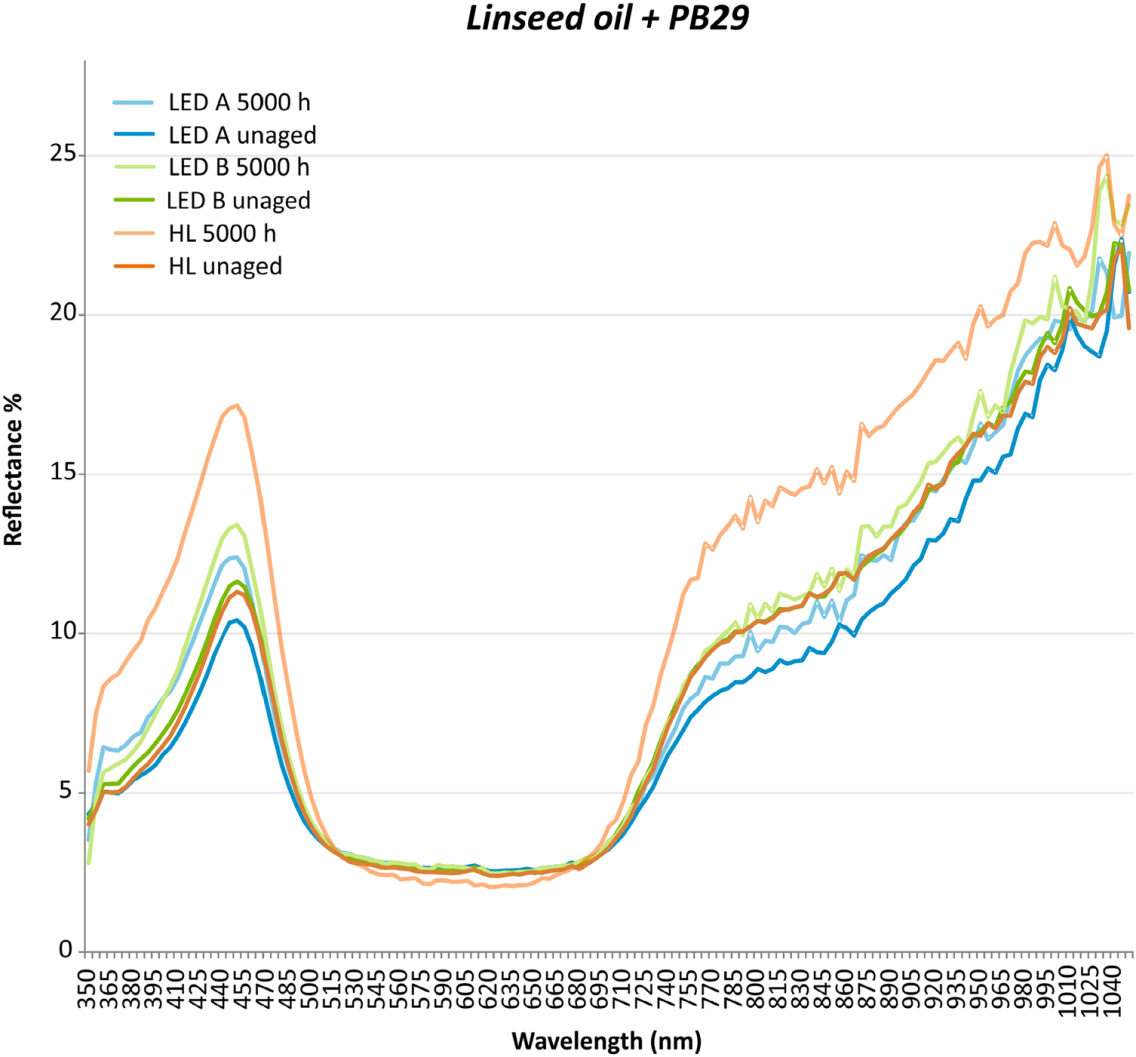
Reflectance spectra of the unaged and 5000 h accelerated light (LED A—420 nm, LED B—460 nm, HL—halogen lamp) aged linseed oil mixed with ultramarine blue PB29.

The UV/Vis/NIR measurements results of all investigated unaged and aged acrylic, alkyd, and linseed oil paint samples are summarized in Table 2, which includes the shift in the values of the lightness/darkness (L*), redness/greenness (a*), yellowness/blueness (b*), and total colour (E*). According to the literature^21^, an inexperienced observer would notice differences in colour for a total colour shift (ΔE*) above 2. By comparing the results between the unaged and aged samples, it is possible to notice that some pigments combined with linseed oil were the least stable. Particularly, the paint sample based on the mixture of linseed oil and ultramarine blue (PB29) showed the largest sensitivity to the three lighting systems. This sample aged under the LED A and halogen lamp had a colour shift towards green and yellow (− a*, + b*), while the average total colour change Δ*E*_ab_* corresponded to 5.32 with standard deviation (SD) of 3.25 and 4.47 with SD of 1.48, respectively. Additionally, the mock-up exposed to the halogen lamp had an increase in reflectance from approximately 11–17 of the maximum band at 450 nm (Fig. 2), which is very likely due to a change of the paint surface roughness during the light exposure^22^. On the other hand, when exposed to LED B the direction of the colour shift was opposite, namely towards red and blue (Δa* = 0.93 ± 3.11 and Δb* = − 2.16 ± 5.10) while ΔE*_ab_ was 4.9 with 3.81 SD.

**Table 2:** Shifts in the lightness–darkness (ΔL*), redness–greenness (Δa*), yellowness–blueness (Δb*) coordinates, and total colour (ΔE*_ab_ 1976) of the 5000 h accelerated light aged (LED A—420 nm, LED B—460 nm, and Halogen—halogen lamp) (a) acrylic, (b) alkyd, and (c) linseed oil-based samples with their averages (Avg) and standard deviations (SD) values obtained with colour measurements.

(a) Acrylic-based mock-up		ΔL* (Avg/SD)	Δa* (Avg/SD)	Δb* (Avg/SD)	ΔE*_ab_ (Avg/SD)
Cadmium yellow PY37	LED A	0.05 ± 0.17	0.00 ± 0.27	− 0.20 ± 0.36	0.45 ± 0.17
	LED B	0.04 ± 0.08	− 0.29 ± 0.11	0.26 ± 0.43	0.49 ± 0.31
	Halogen	− 0.04 ± 0.32	− 0.16 ± 0.50	− 0.12 ± 0.32	0.63 ± 0.13
Cadmium red PR108	LED A	− 0.10 ± 0.13	0.13 ± 0.22	0.11 ± 0.04	0.28 ± 0.15
	LED B	− 0.02 ± 0.19	0.17 ± 0.49	− 0.07 ± 0.29	0.48 ± 0.35
	Halogen	0.56 ± 0.87	− 0.95 ± 0.97	0.75 ± 0.67	1.38 ± 1.42
Chrome green PG18	LED A	0.22 ± 0.08	− 0.07 ± 0.05	− 0.05 ± 0.07	0.25 ± 0.09
	LED B	− 0.09 ± 0.13	0.09 ± 0.26	− 0.07 ± 0.17	0.32 ± 0.13
	Halogen	0.05 ± 0.03	0.17 ± 0.14	− 0.11 ± 0.07	0.23 ± 0.13
Ultramarine blue PB29	LED A	0.05 ± 0.15	− 0.06 ± 0.12	0.21 ± 0.10	0.28 ± 0.09
	LED B	− 0.09 ± 0.06	0.11 ± 0.16	0.05 ± 0.18	0.25 ± 0.10
	Halogen	− 0.07 ± 0.03	− 0.05 ± 0.14	0.16 ± 0.27	0.28 ± 0.19

(b) Alkyd-based mock-up		ΔL* (Avg/SD)	Δa* (Avg/SD)	Δb* (Avg/SD)	ΔE*_ab_ (Avg/SD)
Cadmium yellow PY37	LED A	0.03 ± 0.12	− 0.41 ± 0.09	− 0.07 ± 0.27	0.48 ± 0.12
	LED B	0.05 ± 0.13	− 0.41 ± 0.11	0.05 ± 0.09	0.43 ± 0.14
	Halogen	0.01 ± 0.09	− 0.37 ± 0.05	− 0.24 ± 0.22	0.47 ± 0.15
Cadmium red PR108	LED A	0.19 ± 0.05	− 0.01 ± 0.29	0.00 ± 0.20	0.37 ± 0.08
	LED B	0.15 ± 0.09	0.01 ± 0.18	− 0.05 ± 0.12	0.24 ± 0.13
	Halogen	0.26 ± 0.06	− 0.06 ± 0.09	0.00 ± 0.07	0.29 ± 0.06
Chrome green PG18	LED A	0.17 ± 0.20	0.10 ± 0.13	0.05 ± 0.09	0.28 ± 0.13
	LED B	0.07 ± 0.05	0.10 ± 0.08	− 0.06 ± 0.04	0.16 ± 0.06
	Halogen	0.15 ± 0.07	0.20 ± 0.09	− 0.02 ± 0.08	0.28 ± 0.07
Ultramarine blue PB29	LED A	− 0.55 ± 0.13	0.59 ± 0.35	0.07 ± 0.33	0.90 ± 0.24
	LED B	− 0.34 ± 0.09	0.44 ± 0.05	0.20 ± 0.12	0.61 ± 0.05
	Halogen	− 0.80 ± 0.13	1.06 ± 0.29	0.34 ± 0.25	1.38 ± 0.36

(c) Linseed oil-based mock-up		ΔL* (Avg/SD)	Δa* (Avg/SD)	Δb* (Avg/SD)	ΔE*_ab_ (Avg/SD)
Cadmium yellow PY37	LED A	0.22 ± 0.17	− 0.68 ± 0.17	− 0.64 ± 0.48	1.01 ± 0.41
	LED B	0.11 ± 0.20	− 0.75 ± 0.23	− 1.65 ± 1.15	1.85 ± 1.12
	Halogen	0.06 ± 0.08	− 0.51 ± 0.09	− 0.64 ± 0.04	0.86 ± 0.35
Cadmium red PR108	LED A	− 0.14 ± 0.05	0.56 ± 0.19	0.55 ± 0.15	0.81 ± 0.18
	LED B	0.25 ± 0.11	0.08 ± 0.38	− 0.28 ± 0.59	0.68 ± 0.34
	Halogen	− 0.01 ± 0.14	0.43 ± 0.37	0.36 ± 0.13	0.59 ± 0.35
Chrome green PG18	LED A	0.95 ± 0.25	0.91 ± 0.35	− 0.01 ± 0.06	1.32 ± 0.40
	LED B	1.62 ± 0.35	2.23 ± 0.82	0.29 ± 0.18	2.79 ± 0.85
	Halogen	2.04 ± 0.23	3.01 ± 0.40	0.28 ± 0.13	3.65 ± 0.45
Ultramarine blue PB29	LED A	− 0.16 ± 0.94	− 2.53 ± 2.28	4.05 ± 3.36	5.32 ± 3.25
	LED B	− 0.23 ± 1.38	0.93 ± 3.11	− 2.16 ± 5.10	4.90 ± 3.81
	Halogen	− 0.18 ± 0.25	− 2.64 ± 0.96	3.59 ± 1.17	4.47 ± 1.48

The high SD values can be explained by the two main components used for preparing the specimens such as the binder and the pigment. Although the mixing of the binder and pigment was properly performed, the drying process of the linseed oil caused the formation of colour unevenness on the paint surface, which was easy recognizable by naked eye and also explained by the obtained high SD values (Table 2). In order to elucidate the reason of the major colour change of the linseed oil—ultramarine blue PB29, supplementary analyses are necessary (e.g. THM-GC/MS, µ-FTIR). These will be the subject of a near future publication.

Another type of linseed oil-based sample that proved to be sensitive to the ageing was the one containing chrome green (PG18). In this case the halogen and LED B lights proved to be the most harmful, demonstrated by ΔE*_ab_ of 3.65 ± 0.45 and ΔE*_ab_ of 2.79 ± 0.85, respectively. This colour change is mostly based on a shift towards red (Δa* = 3.01 ± 0.40) and an increase in brightness (ΔL* = 2.04 ± 0.23). Furthermore, a higher reflectance varying from 7 to 10% of the maximum band at 385 nm was also recorded (see Supplementary Fig.S1 online).

In contrast to the linseed oil-based paint mock-ups, the acrylic and alkyd-based paints did not show any significant shift in colour (Table 2a,b), thus resulting the most stable. The higher colour shift of the linseed oil samples can be partially attributed to the binder component according to the colour measurements performed on the unaged and aged pure binders. The pure oil binder showed a greater sensitivity to the light systems in comparison to the acrylic and alkyd binder (see Supplementary Table S2 online). Because the surface of the binder specimens was shiny, the reflectance of these samples was measured with specular component inclusion (RSIN) in addition to the specular component exclusion (RSEX). There is no contradiction between the measurement results made by the two specular components.

#### Multivariate analyses

##### Exposure time

**Figure 3:**
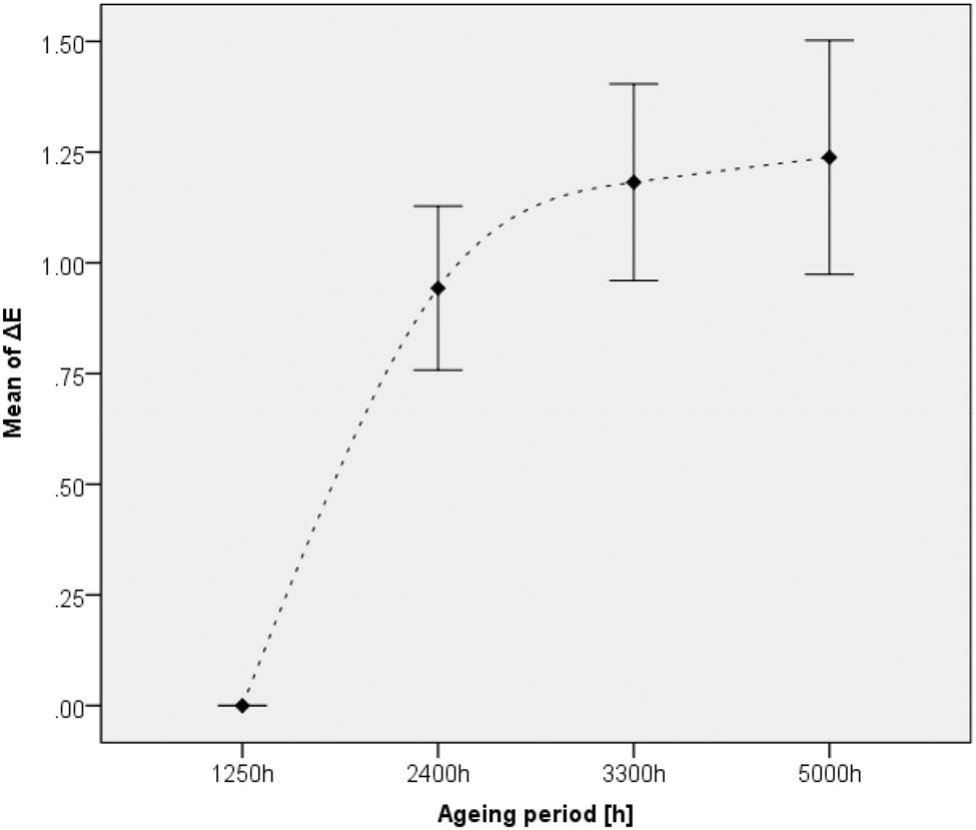
Correlation between colour change (mean of ΔE) and ageing period (hours).

The connection between the CIELAB ΔE*_ab_ colour change and the exposure time was investigated using variance analysis. This method proved a significant relationship between these variables based on the correlation coefficient *r* = 0.616 and significance level *p* < 0.05. Correlation coefficient *r* indicates the strength and direction of correlation, while *p* represents the presence or absence of the relationship, where being the *p* value closer to zero the more significant the connection is. As depicted in Fig. 3 variance analyses showed not only that the most significant total colour change occurred after 5000 h of ageing—also in accordance with the univariate analyses—but also that the ageing process slowed down after 2400 h of exposure. The measurement points shown in this figure were obtained as results of a total of 360 measurements carried out at each stage of ageing. The figure shows an increasing deviation with ageing time, which is the consequence of the different ageing characteristics of stable and unstable samples. The magnitude of colour change depending on pigment type is explained in detail by Table 2.

##### Binding medium of the mock-ups

**Figure 4:**
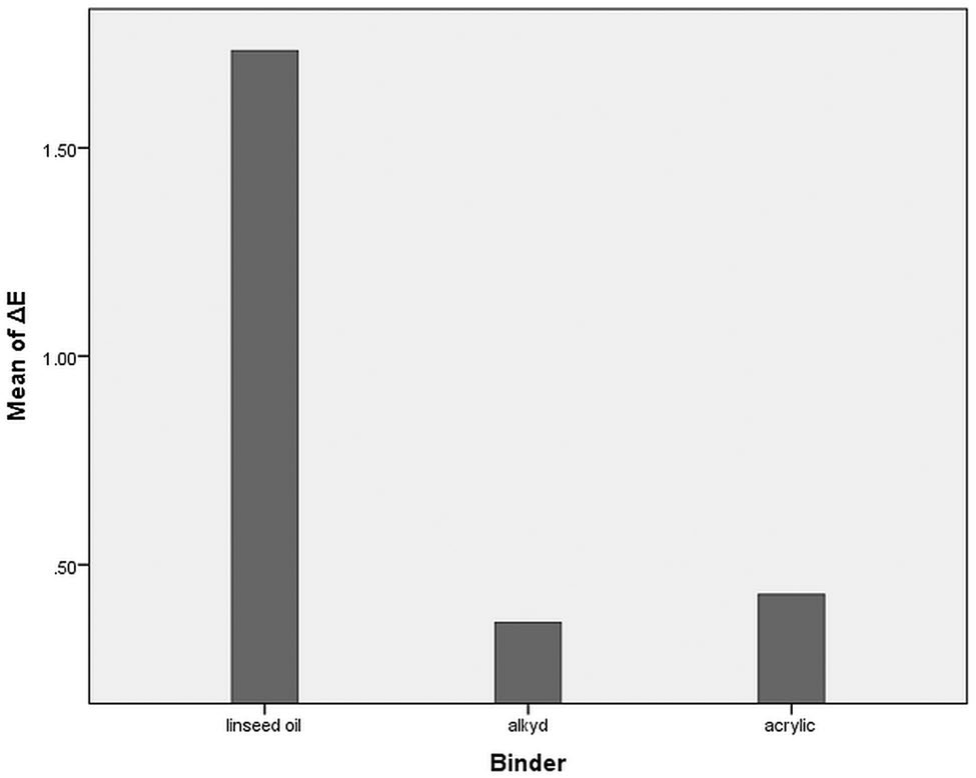
Correlation between colour change (mean of ΔE) and binding medium used for the mock-ups (acrylic, alkyd, and linseed oil).

When comparing ΔE*_ab_ and the type of binder used for the mock-ups it is possible to observe a noteworthy relationship between them (*r* = − 0.301 and *p* < 0.05). This association highlights the largest change in colour of linseed oil-based mock-ups compared to the other two binding materials such as acrylic and alkyd (Fig. 4). On the other hand, the alkyd and acrylic mock-ups can be considered stable.

##### Lighting system

**Figure 5:**
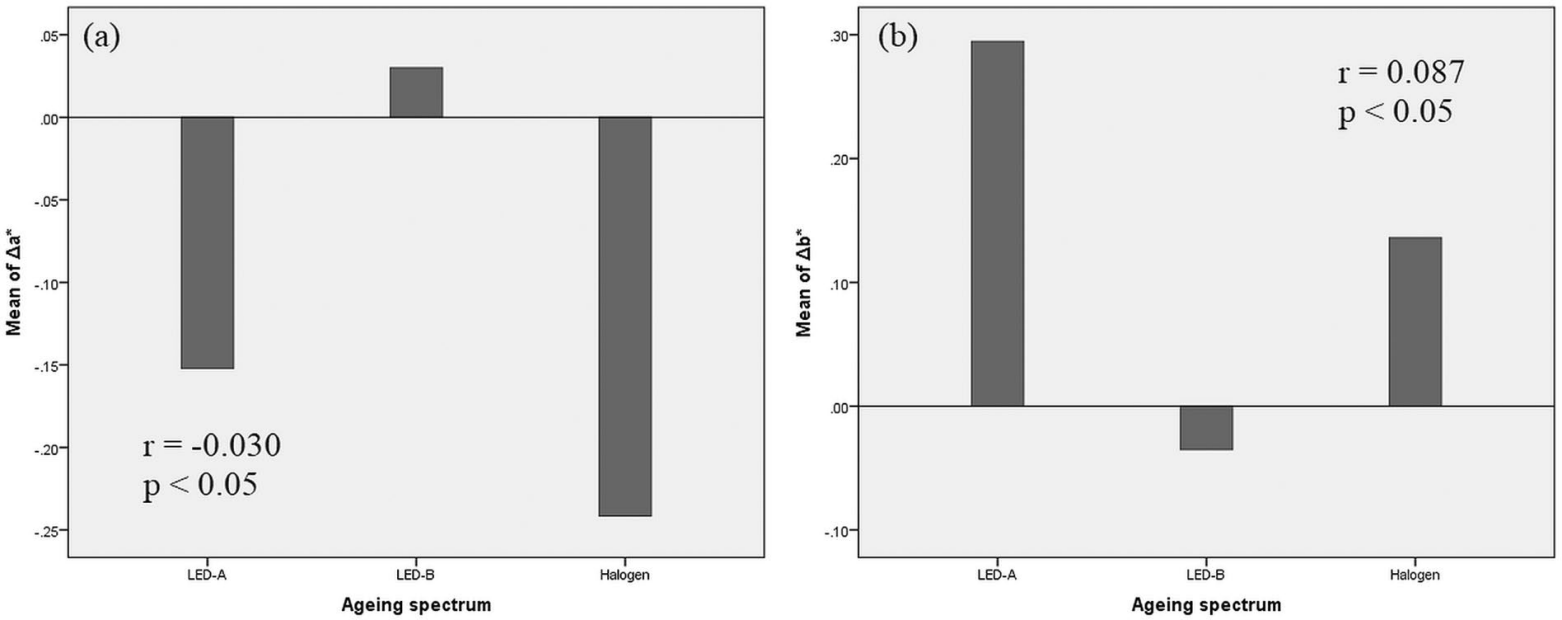
Correlation between (**a**) CIELAB Δa* (red–green), (**b**) CIELAB Δb* (yellow–blue) and lighting systems (LED A—420 nm, LED B—460 nm, HL—halogen lamp).

Based on the higher significance level of *p* > 0.05 no significant relationship between the variables of ΔE*_ab_ and lighting type as well as between ΔL* lightness and lighting system could be observed. This result suggests that the overall degree of colour change ΔE*_ab_ observed in the samples took place during the ageing regardless the type of lighting system used. In contrast, there is a weak (r < 0.1) but significant relationship between the colour shift along the colour axes (CIELAB a* and b*) and the lighting type. As can be observed in Fig. 5, the LED A and halogen lamp caused the biggest colour shift along both colour axes a* and b*, while in case of LED B the mean values do not show any major change. More precisely, along the red–green a* axis the greatest colour shift is given by the halogen lamp, in which the red wavelength component dominates (Fig. 1c). On the other hand, the greatest change along the yellow–blue b* axis was generated by the LED A, in which the blue wavelength component is prevalent (Fig. 1a). According to these results, the colour shift along the colour axes is dependent on the dominant wavelength of the used lights sources.

##### Pigment of the mock-ups

**Figure 6:**
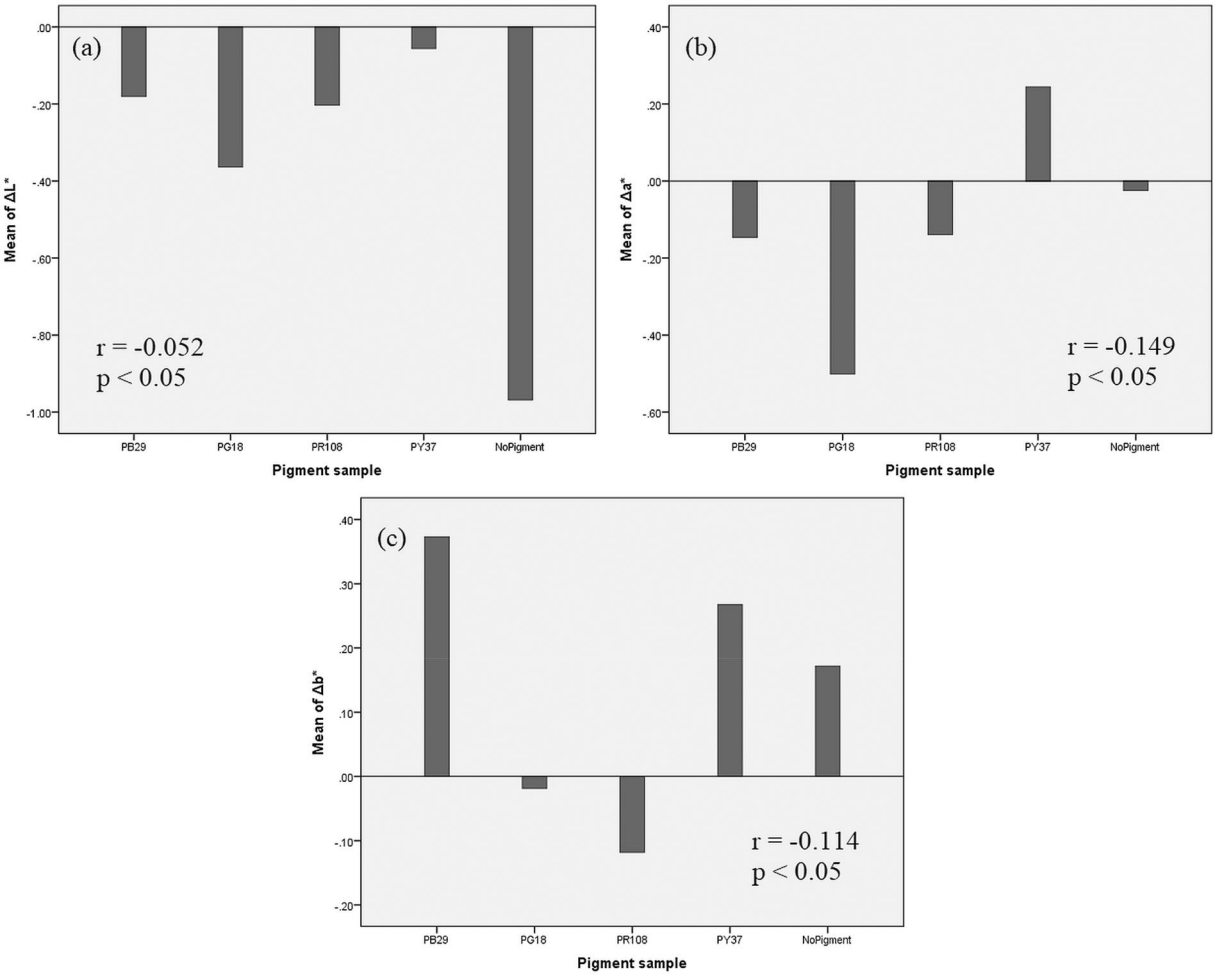
Correlation between colour change (mean of ΔE) and pigment type used for the samples (PB29 = ultramarine blue/PG18 = chrome green/PR108 = cadmium red/PY37 = cadmium yellow), and binders without pigments (NoPigment): along CIELAB (**a**) L*, (**b**) a*, and (**c**) b* axes.

The type of pigment used for the paint sample plays a significant role (*r* = 0.067 and *p* < 0.05) in the degree of colour shift. Generally, samples containing the ultramarine blue PB29 pigment and binders without pigment show the greatest ΔE*_ab_ (see Supplementary Figs. S2 and S3 online). Examining the shifts of the main colour coordinates along the CIELAB L*, a*, and b* axes, it is possible to observe that the change in lightness L* (Fig. 6a) is more significant for samples not containing pigments, while the chrome green PG18 specimens have the slightest shift in lightness among all paint samples. On the other hand, it is possible to state that the colour shift along a* and b* colour axes (Fig. 6b,c) strongly depends on the colour of the sample. For instance, the biggest changes along the yellow–blue b* axis occurred principally to the yellow or blue samples (cadmium yellow PY37 and ultramarine blue PB29), while along the red–green a* axis the green samples (chrome green PG18) showed the most prominent differences.

## Conclusion

The effect of two different new developed LED systems for indoor museums—one having the maximum blue peak at 420 nm and the second one at 460 nm—and a traditional incandescent halogen lamp on self-made modern paints was first investigated by UV/Vis/NIR spectrophotometry. The results obtained in this research clearly highlight the higher sensitivity of the linseed oil binder combined with the ultramarine blue PB29 pigment towards the lights exposure in comparison to all paint samples. Univariate analyses demonstrated that this combination of binder and pigment had the highest colour shift (ΔL*, Δa*, and Δb*) and greatest total colour change ΔE*_ab_, which were more prominent after 5000 h, while the acrylic and alkyd mock-ups remained stable. In agreement with these results, multivariate analyses such as variance analyses evidenced the strong relationship between the type of binder and pigment used for the mock-ups such as linseed oil and ultramarine blue PB29 with the strongest variation in colour and exposure time. Indeed, the degree of colour change increased with the ageing process showing the biggest change at 2400 h. After that the ΔE*_ab_ slowed down till reaching the maximum at 5000 h. Furthermore, contrary to the effect of the binding materials and the inorganic pigments on the colour shift (ΔL*, Δa*, and Δb*), variance analyses showed that there is not a significant relationship between the variables of CIELAB ΔE*_ab_ colour change and lighting systems. Accordingly, the spectrum of light also does not affect the degree of overall CIELAB ΔE*_ab_ colour change. However, there is a weak, but significant relationship between the colour shift along the colour axes (CIELAB a* and b*) and the illuminants, which shows the dependence of the shift in the colour axes of the samples with the dominant wavelength of the light source. For instance, the halogen lamp caused the greatest colour shift along the red–green a* axis, while along the yellow–blue b* axis the LED A generated the largest change. Moreover, variance analyses demonstrated that the colour shift along the colour axes depends not only on the dominant wavelength of the light source (red for halogen lamp and blue for LED A) but also on the colour of the sample. Indeed, the highest shift along the yellow–blue axis was shown by the cadmium yellow PY37 and ultramarine blue PB29 mock-ups, while the most prominent shift along the red–green axis was represented by the chrome green PG18 samples. The results obtained in this work will be used for studying a correlation between the detected colour changes and the chemical stability of the investigated samples. For this purpose, further scientific investigations will be employed, and reported in a near future article.

## Methods

### Materials

The selection of materials for this research was aimed to reproduce as much as possible paint colours used in the field of modern and contemporary art and thus were mostly based on different types of synthetic organic binders and pigments. The class of inorganic pigments was chosen for the preparation of the samples allowing for a better and deeper focus the research on only one class of pigments. Thus, “2-components self-made paint” mock-ups were prepared by mixing four different inorganic pigment (P) powders (Kremer Pigmente, Germany) such as ultramarine blue PB29, chrome green PG18, cadmium red PR108, and cadmium yellow PY37 with an alkyd (Medium 4—Lukas, Germany), acrylic (Plextol D498—Kremer Pigmente, Germany) and linseed oil (Kremer Pigmente, Germany) binding media (BM) in different ratio (P/BM) depending on the consistence of the paint achieved. A detailed description of the mock-ups can be found as Supplementary Table S1 online. The paints were applied on microscope glass slides with a thickness of 150 µm, and left drying at room conditions for eight weeks. The same procedure was used to prepare mock-ups of pure binding media, without addition of pigments.

### Lighting systems

For the lighting exposure of the samples three different lighting systems were used. Two were based on spectrally tuneable LED light sources, installed in lighting booths. Each of these LED luminaires has 20 different channels with separated LED peak wavelengths between 414 and 691 nm. Among the total channels, 17 are monochromatic LEDs—mainly Golden Dragon series LEDs of OSRAM, Germany—while three channels are composed of white phosphor LEDs—Oslon series LEDs of OSRAM, Germany. The Spectral Power Distribution (SPD) of each LED channel of the luminaire is illustrated as Supplementary Fig.S4 online. The SPD describes the energy emitted by each source at different wavelength of the considered electromagnetic spectrum between 380 and 730 nm, thus determining how the light appears and how paint colours are rendered. Each colour channel of the luminaire contains 24 LEDs from the same type, thus resulting in a total of 480 LEDs in one spectrally tuneable LED luminaire. Furthermore, the third lighting booth contained a set of cold mirror incandescent halogen lamps, which is a widely used light source in museum environment. As it can be seen in Supplementary Fig.S5 online, the spectral range of each used lighting system differed from each other in spectral composition. The main dissimilarity between the LED spectra was the spectral content below 500 nm. The spectral range named as LED A included a maximum peak of the blue range at 420 nm, while the spectral range named as LED B contained a maximum peak of the blue range at longer wavelength as 460 nm, while the short wavelength emission was minimized. The blue peak wavelengths for the two ageing spectra were specifically chosen to be the minimum and maximum practically applicable blue wavelength emission content in a museum environment in order to investigate the effects of these extreme cases. On the other hand, the halogen incandescent lamp was characterized by a spectral field that included a small amount of infrared radiation. Prior the light exposure ageing of the samples, the uniformity of illumination was measured in all of the three ageing booths by using the portable SPIC-200 spectral irradiance colorimeter (EVERFINE Corporation, China) on several different points occupied by the samples.

The above described three lighting systems were chosen for this project to determine the effect of their electromagnetic spectral content on photo-oxidation processes of art materials. Additional interest was paid on to investigating whether the lighting booth with short wavelength blue LED could cause more significant changes on the surface of the paint material as well as in the paint structure on the basis of the CIE publication and other previous studies^23^.

### Colour parameters of the lighting systems

The main colorimetric parameters that define how the colour of the light source appears were the Correlated Colour Temperature (CCT) and *d*_UV_. The CCT explains how cool (bluish) or warm (yellowish) nominally white light looks like, thus characterizing the appearance of the emitted light and not of the colour of the exposed objects. By considering that the colour appearance of a hypothetical “black body” varies from dull cherry red (described as warm), then glowing orange–red, to eventually bright white-hot (define as cool) as its temperature increases by getting hotter when irradiated by a light source, the CCT is the absolute temperature of a black body given in kelvin (K) when its emitted light most closely matches the colour appearance of the light source^24^. The CCT of a white light source is normally distinguished in intermediate—between 3300 and 5000 K—warm or yellowish—below 3300 K – and cool or bluish—higher than 5000 K^24^. So far specifications in indoor museums tended to opt for a 3000 K CCT^25^, mostly because LEDs with higher CCT are considered to have an unacceptably large peak in the “blue region” of the spectrum^1^. Concerning this last observation, it has to be considered that the Spectral Power Distribution (SPD) of two different light sources with the same CCT value can be different^2^, thus making the CCT a generic number. To describe this diversity, the *d*_UV_ parameter has been introduced by the American National Standards Institute (ANSI)^26^. It quantifies the distance between the chromaticity of a given light source and a black body of both equal CCT. A negative *d*_UV_ indicates that the source has a purplish tone being “below” the black body locus, while a positive *d*_UV_ shows that the source has a greenish tone being “above” the black body locus. Lamps with a positive *d*_UV_ (greater than 0.006) are suggested to be avoided because they may introduce a greenish appearance^1^. Together, a specific CCT value and *d*_UV_ value correspond to a specific pair of chromaticity coordinates. For this study the CCTs of the three lighting systems were approximately 3700 K (neutral white light), whereas the *d*_UV_ was similarly close to 0 (Table 1).

### Colour rendition of the lighting systems

Another important factor of a light source is its rendering ability or Colour Rendering Index (CRI) expressed by *R*_a_, which defines the ability of a light source to make appear an object as “natural” as possible. A CRI- *R*_a_ close to 100 is “best or true” while those with a CRI > 80 are considered good. Nowadays, a minimum CRI-*R*_a_ of 85 seems to be used for indoor museums and galleries while the best quality LEDs offers a CRI-*R*_a_ > 90 to ensure vibrant red colours^18^. Within this project the used test lights had a high CRI-*R*_a_^19^ (≥ 88) (Table 1). Additionally, according to the Technical Memorandum (TM) 30–15 of the Illuminating Engineering Society (IES)^27^ the colour fidelity *R*_f_ and the gamut area *R*_g_ index were calculated (*R*_f_ ≥ 79 and *R*_g_ ≥ 97, Table 1) for evaluating effectively the colour rendering of the used light sources. Analogous to CRI-Ra, the colour fidelity *R*_f_ index characterizes the average difference in colour for several Colour Evaluation Samples (CES) (99 in case of the IES TM-30 *R*_f_^27^ and 8 for the CIE-*R*_a_^19^) by comparing the appearance of the samples under the test and reference conditions. In this way within a range from 0 to 100, *R*_f_ value of 100 indicates a perfect match with the reference. On the other hand, the gamut area *R*_g_ index indicates the average level of saturation relative to the same CCT reference illuminant, in which a value of 100 indicates the same average gamut area. *R*_g_ < 100 corresponds to an average decrease of saturation while an average increase of saturation goes to *R*_g_ > 100. However, the shape of the gamut of two light sources with the same *R*_g_ value can be different.

## Supplementary Information

Supplementary Information.

## Data Availability

The most significative data generated or analysed during this study are included in this published article (and its Supplementary Information files). Further results obtained during the current study are available from the corresponding author on reasonable request.
