# Supplementary material for: The effect of new LED lighting systems on the colour of modern paints
Source: Sci Rep. 2021 Nov 17;11:22375. doi: 10.1038/s41598-021-01836-9 (PMC8599686; doi:10.1038/s41598-021-01836-9)
Supplement: Supplementary file 1 — Supplementary Information. [file 41598_2021_1836_MOESM1_ESM.docx]

**The effect of new LED lighting systems on the colour of modern paints**

Valentina Pintus^1,2*^, Ferenc Szabó^3^, Renáta Gazdag-Kéri^3^, Dávid Noel Tóth^3^, Róbert Nagy^3^, Péter Csuti^3^, Katja Sterflinger^1^, Manfred Schreiner^1^

1. Institute of Science and Technology in Art, Academy of Fine Arts, Schillerplatz 3, 1010 Vienna, Austria
2. Institute for Conservation-Restoration, Modern-Contemporary Art, Academy of Fine Arts, Schillerplatz 3, 1010 Vienna, Austria
3. Light and Colour Science Research Laboratory, Faculty of Information Technology, Department of Electrical Engineering and Information Systems, University of Pannonia, Egyetem Str., Veszprém, Hungary

Corresponding author´s email: [v.pintus@akbild.ac.at](mailto:v.pintus@akbild.ac.at)

**Experimental**

**Lighting chambers set-up**

In order to expose in a proper way, the self-made paint samples to the two new developed LED lighting systems and to the halogen incandescent lamp the construction of a chamber for each respective light source was performed. **Figure S6** shows the set-up of the lighting booths used for the accelerated ageing experiments. The assembling of the ageing chambers was slightly different in case of the LED based booths and the halogen incandescent one. While the LED based ageing chambers were closed from all sides, the halogen booth was opened at the top of the box **(Fig.S6)**. Thus, the heat emitted by halogen lamp could escape from the ageing chamber and the damaging effects of heat could be minimized.


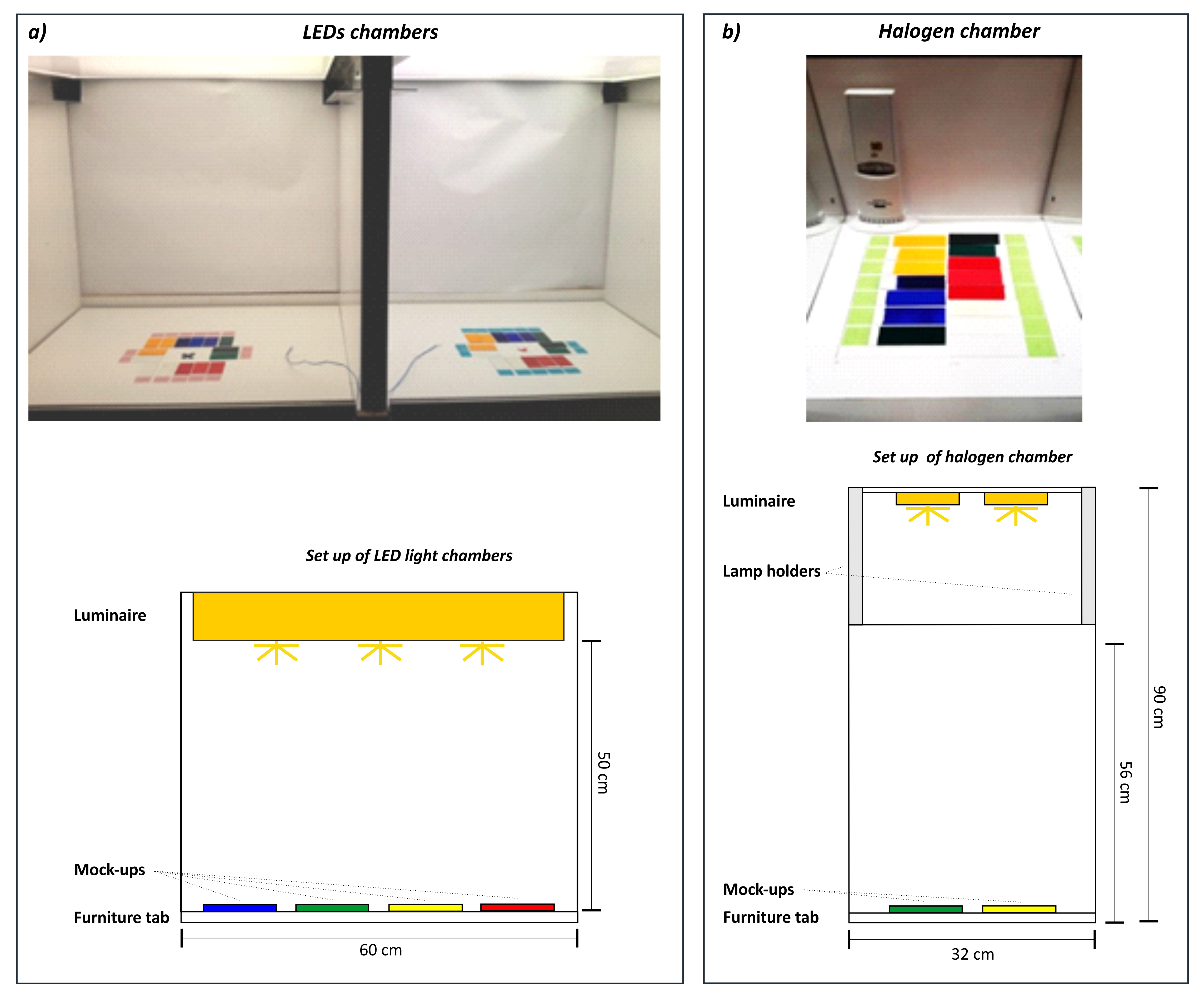


*Fig.S6: Images of the three build up lighting ageing chambers containing the paint samples and their respective plan forms:* ***a)*** *LEDs and* ***b)*** *halogen lamp.*

The other difference between the ageing chamber constructions is the distance between the luminaire and the paint samples. While this distance in the LEDs based lighting booths was 50 cm, in the halogen booth it corresponded to additional 40 cm for a total of 90 cm to compensate the amount of heat emitted by the halogen light source. Additionally, thin lamp holders with large openings were incorporated at the top of the halogen booth for decreasing the amount of the accumulated heat **(Fig.S6b)**.

As can be seen in **Fig.S6a)** and **b)** the light sources are placed relatively close to the samples - 50 cm and 90 cm - compared to indoor museums. It serves to the purpose of speeding up the ageing process by increasing the dose of radiation, thus making the experiment feasible in the given time limits of the performed research project. Test samples were placed on white furniture tabs as supports and for avoiding the possibility of overheating during the exposure. During the artificial ageing process, the ageing of the lighting systems was also considered; the spectral output was measured twice a week, and corrected when necessary to ensure a constant ageing spectrum. All measurements of the spectral output of the ageing chambers were carried out on the stabilized operating temperature of the luminaires. Blue light leakage due to the failure of the phosphor LEDs could not be noted during these control measurements.

**Ambient temperature (T °C) and relative humidity (RH %)**

The three light booths were placed in an air-conditioned dark laboratory room (T 22 °C ± 1 °C). During the ageing process the temperature and relative humidity inside the lighting chambers were continuously monitored, which corresponded to 25 ± 3 °C and 15 to 30 % RH, respectively. The monitoring of the temperature and relative humidity data in the LED ageing chambers was performed with the TC-301 2-channel temperature instrument (Dostmann electronic GmbH, Germany), while in the halogen booth the data were collected with the TA120 temperature station (TFA Dostmann GmbH & Co. KG). Furthermore, the LED light sources on top of the chambers were cooled by fans in order to guarantee stable spectral output during the ageing.

**Exposure time**

For the investigations of the LED and halogen lamp effect on the colour of modern paint materials UV/Vis/NIR was used.

The paint samples were analysed before the beginning of the exposure under the lighting systems as unaged specimens and also at certain intervals of time along the total exposure of 5000 hour (as the maximum threshold exposure or critical radiation time t_s_) in order to monitor any possible colour and chemical change occurring in the paint. The intervals of time corresponded to 1250, 2400, 3300, and 5000 hours.

Each lighting system reached the threshold exposure at different periods. For instance, the maximum 5000 hours irradiance period was reached by the halogen lamp corresponding to 138 % of total threshold dose, while for the LED B (460 nm) it was 330 % and for LED A (420 nm) it was 439 % **(Tab.S3)**. On the other hand, LED A samples reached the threshold value before the first reflectance measurement (after 1146 hours irradiance dose, **Tab.S3**), while the specimens exposed under LED B reached the critical dose during the second ageing period (after 1520 hours irradiance dose, **Tab.S3**). Furthermore, samples exposed to the halogen lamp achieved the threshold dose only during the last ageing period, after 3532 hours irradiance dose. This is mostly because halogen spectrum contains less radiation in the harmful spectral range (420 nm – 450 nm) compare to the LED ageing spectra.

| ***Part of total threshold dose (%)*** | | | | |
| --- | --- | --- | --- | --- |
| **Lighting system** | **Exposure time** | | | |
|  | 1250 h | 2400 h | 3300 h | 5000 h |
| LED A | **112%** | 211% | 293% | 439% |
| LED B | 84% | **159%** | 221% | 330% |
| Halogen | 35% | 67% | 93% | **138%** |

*Tab.S3: List of the part of the threshold dose (%) reached during the exposure periods (hours) under the LED A (420 nm), LED B (460 nm), and Halogen (halogen lamp).*

**Colour change detection**

**Ultraviolet / Visible / Near Infrared (UV/Vis/NIR) spectrophotometry**

Ultraviolet / Visible / Near Infrared (UV/Vis/NIR) measurements of the samples were conducted with the UltraScan Pro Sensor (HunterLab, Germany) spectrophotometer using a D65 illuminant in the range of 350 nm - 1050 nm. Reflection spectra were measured relative to the white standard of the instrument and a 2° standard observer. The spectrophotometer was set to specular component excluded (RSEX) measurement configuration because of the matte surface of the samples. Conversely, the pure organic binders were also measured with the specular component included (RSIN), because the layer of these samples was shiny. The samples were measured in 5 total pre-set positions in order to have representative and reproducible measurement results from each sample. These pre-set positions were obtained by developing a custom-made positioning template to support the analyses on the same exact spot at different measuring times but also for the accurate repositioning of the samples for each measurement. The smallest (7 mm diameter) port plate in standard diffuse/8° geometry was used for the analyses.

The obtained results are distinguished in those evaluated by considering one variable at time (e.g. shift of the colour coordinates, total colour change, etc.), which are by definition the *univariate analyses*, and those calculated by considering two or more variables (e.g. total colour changes with binder or pigment type, etc.) known as *multivariate analyses*, which are hereby described.

Univariate analyses

For the univariate analyses, the *L** (lightness-darkness), *a** (redness-greenness), and *b** (yellowness-blueness) polar coordinates of the CIELAB colour space system (Commission Internationale de l´Eclairage) were used for the identification of the paint samples. For the lighting ageing studies, the colour change of the specimens was calculated by using the total colour difference values (Δ*E**) according to the CIE 1976 (Δ*E**_ab_).

Multivariate analyses

In order to examine the UV/Vis/NIR results more in detail and to investigate the effect of different factors (e.g. ageing time, binder, pigment, as well as lighting system) on colour change, variance analysis was used with one-way ANOVA.


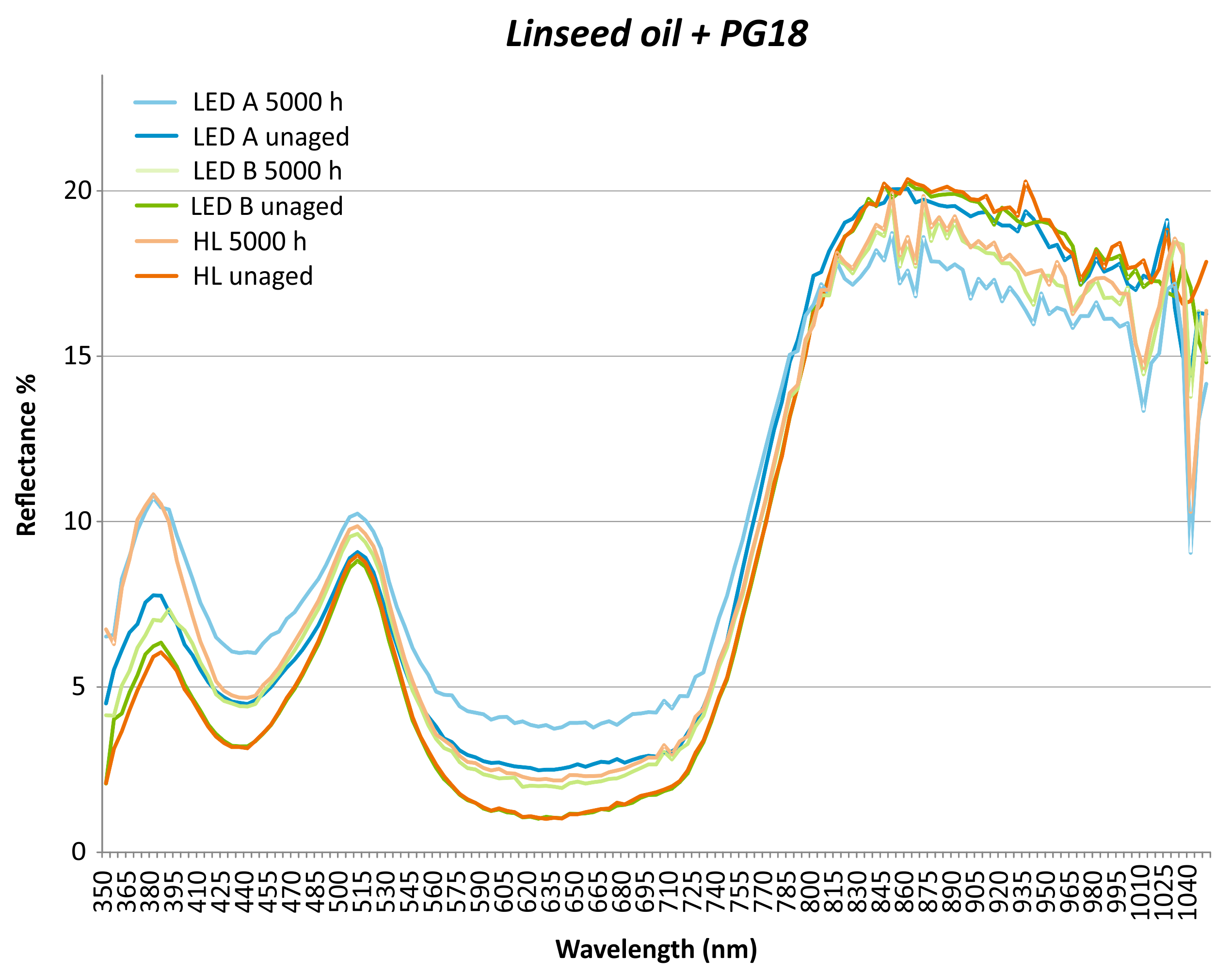


*Fig.S1: Reflectance spectra of the unaged and 5000 hours accelerated light (LED A – 420 nm, LED B – 460 nm, HL – halogen lamp) aged linseed oil mixed with chrome green PG18.*


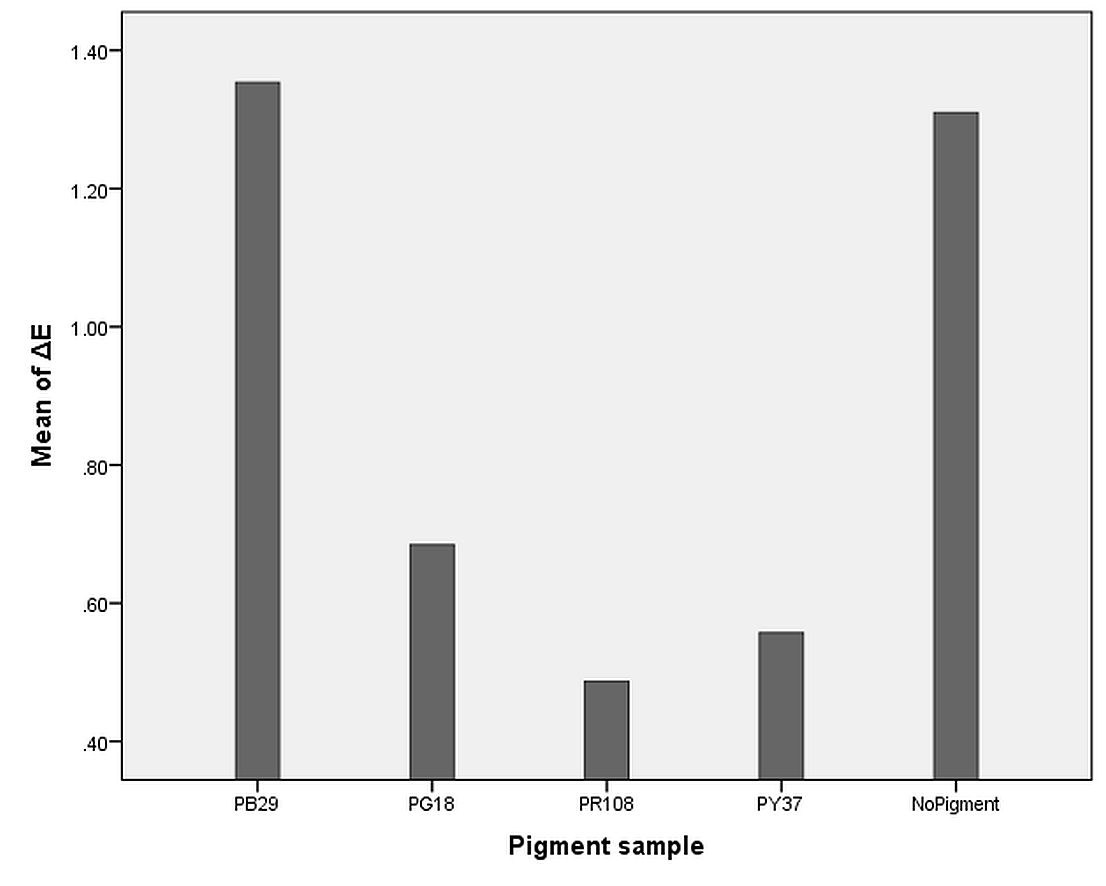


*Fig.S2: Correlation between colour change (mean of ΔE) and pigment type used for the samples (PB29 = ultramarine blue / PG18 = chrome green / PR108 = cadmium red / PY37 = cadmium yellow), and binders without pigments (NoPigment).*


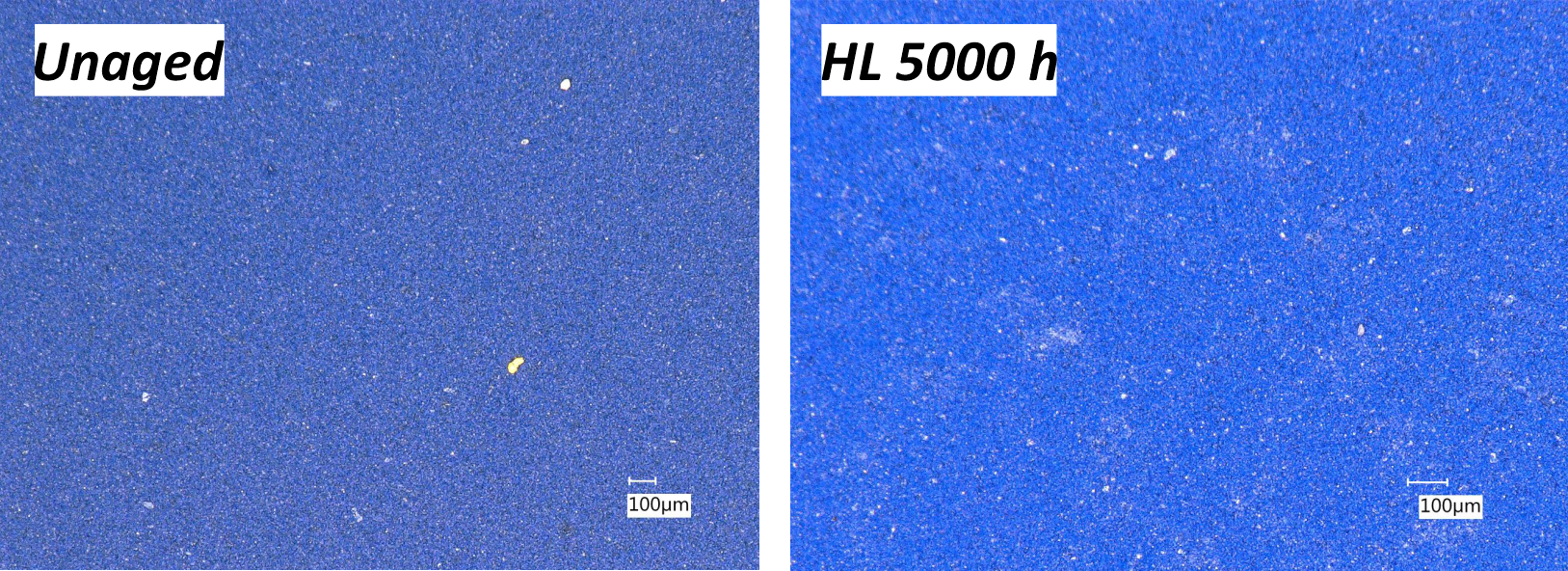


*Fig.S3: Photomicrographs of the Unaged and HL 5000 h aged linseed oil mixed with ultramarine blue PB29* *taken under a VHX-6000 digital microscope (RZ 100x-1000x objective - VH-Z100R Keyence, Japan) with a LED (light emitting diode) light source characterized by a colour temperature of 5700 K. (HL = halogen lamp).*


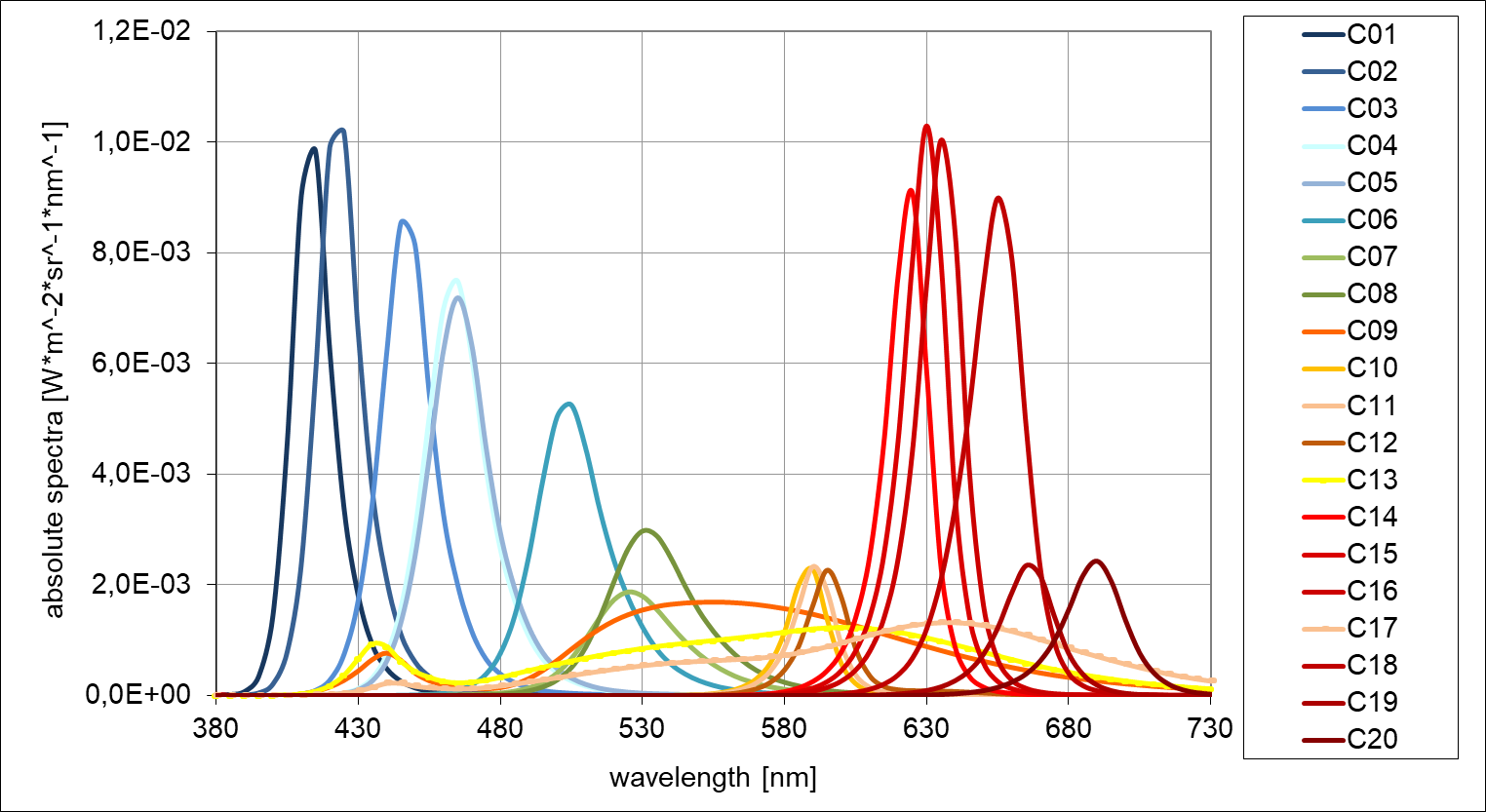


*Fig.S4: LED channel Spectral Power Distribution (SPD) of the tuneable lighting booths.*


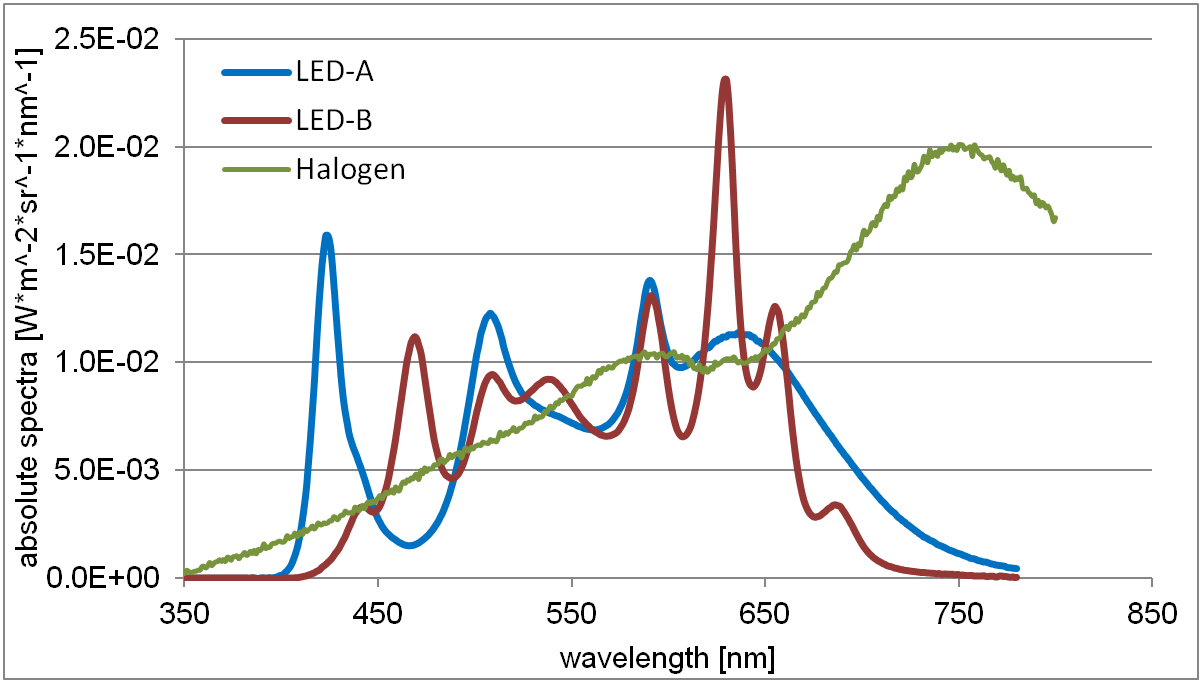


*Fig.S5: Spectral range of the used three types of lighting system such as LED A (420 nm), LED B (460 nm), and halogen lamp.*

| **Pigment Name** | **Chemical composition** | **C.I. Name** | *Alkyd paints*  **P/BM** | |  | *Acrylic paints*  **P/BM** | | *Oil paints*  **P/BM** | |
| --- | --- | --- | --- | --- | --- | --- | --- | --- | --- |
| *Ultramarine blue* | Sulfur/sodium aluminium silicate | PB29 |  | 1:0.7 | | | 1:3 | 1:0.6 |  |
| *Chrome green* | Hydrated chromium oxide | PG18 |  | 1:1.5 | | | 1:1.2 | 1:1 |  |
| *Cadmium red* | Cadmium sulfide/Cadmium selenide | PR108 |  | 1:0.4 | | | 1:3 | 1:0.4 |  |
| *Cadmium yellow* | Cadmium sulfide | PY37 |  | 1:0.7 | | | 1:0.4 | 1:0.5 | |

*Tab.S1: List of self-made mock-up paints containing different pigments. Ratio pigment/binding medium (P/BM) in g/g used for the preparation of the self-made paints is given.*

| **Binder type** | **Lighting type** | **Specular component type** | **Δ*L** (Avg / SD)** | **Δ*a** (Avg / SD)** | **Δ*b**  (Avg / SD)** | **Δ*E** _ab_ (Avg / SD)** |
| --- | --- | --- | --- | --- | --- | --- |
| Acrylic | LED A | RSEX | 0.95 ± 1.34 | 0.05 ± 0.20 | -0.10 ± 0.83 | 1.31 ± 1.16 |
|  |  | RSIN | 0.12 ± 0.09 | 0.03 ± 0.12 | 0.04 ± 0.12 | 0.20 ± 0.04 |
|  | LED B | RSEX | 1.05 ± 0.37 | 0.03 ± 0.70 | -0.19 ± 0.34 | 1.10 ± 0.37 |
|  |  | RSIN | 0.19 ± 0.15 | -0.05 ± 0.10 | 0.04 ± 0.08 | 0.24 ± 0.10 |
|  | Halogen | RSEX | -0.11 ± 0.62 | 0.08 ± 0.36 | -0.17 ± 0.19 | 0.52 ± 0.47 |
|  |  | RSIN | -0.04 ± 0.05 | 0.18 ± 0.03 | -0.03 ± 0.05 | 0.19 ± 0.04 |
| Alkyd | LED A | RSEX | 0.06 ± 0.26 | 0.18 ± 0.11 | -0.39 ± 0.09 | 0.49 ± 0.13 |
|  |  | RSIN | 0.27 ± 0.10 | 0.00 ± 0.05 | -0.04 ± 0.05 | 0.28 ± 0.09 |
|  | LED B | RSEX | 0.55 ± 0.59 | 0.18 ± 0.20 | -0.57 ± 0.22 | 0.95 ± 0.23 |
|  |  | RSIN | 0.04 ± 0.10 | 0.07 ± 0.05 | 0.01 ± 0.05 | 0.12 ± 0.04 |
|  | Halogen | RSEX | 0.29 ± 0.38 | -0.02 ± 0.28 | -0.24 ± 0.54 | 0.54 ± 0.23 |
|  |  | RSIN | 0.21 ± 0.09 | 0.02 ± 0.09 | 0.06 ± 0.07 | 0.24 ± 0.08 |
| Linseed oil | LED A | RSEX | 2.82 ± 3.50 | 0.29 ± 0.09 | -0.47 ± 0.63 | 3.68 ± 2.18 |
|  |  | RSIN | -0.25 ± 0.06 | 0.11 ± 0.03 | 0.03 ± 0.19 | 0.32 ± 0.05 |
|  | LED B | RSEX | 7.27 ± 8.02 | -0.12 ± 0.06 | 1.08 ± 1.05 | 7.36 ± 8.08 |
|  |  | RSIN | -0.28 ± 0.48 | -0.25 ± 0.06 | 0.93 ± 0.05 | 1.08 ± 0.13 |
|  | Halogen | RSEX | 3.61 ± 0.06 | -0.17 ± 0.14 | -0.29 ± 0.72 | 3.69 ± 1.06 |
|  |  | RSIN | -0.25 ± 0.18 | -0.21 ± 0.08 | 0.94 ± 0.14 | 1.01 ± 0.11 |

*Tab.S2: Shifts in the lightness-darkness (ΔL*), redness-greenness (Δa*), yellowness-blueness (Δb*) coordinates, and total colour (ΔE** ***_ab_*** *1976) of the 5000 hours accelerated light aged (LED A – 420 nm, LED B – 460 nm, and Halogen – halogen lamp) acrylic, alkyd, and linseed oil binders with their averages (Avg) and standard deviations (SD) values obtained with colour measurements. The listed values were obtained by with the specular component excluded (RSEX) but also included (RSIN) because of the shiny surface of the samples.*
